# Body mass index vs deuterium dilution method for establishing childhood obesity prevalence, Ghana, Kenya, Mauritius, Morocco, Namibia, Senegal, Tunisia and United Republic of Tanzania

**DOI:** 10.2471/BLT.17.205948

**Published:** 2018-09-10

**Authors:** Adama Diouf, Theodosia Adom, Abdel Aouidet, Asmaa El Hamdouchi, Noorjehan I Joonas, Cornelia U Loechl, Germana H Leyna, Dorcus Mbithe, Thabisile Moleah, Andries Monyeki, Hilde Liisa Nashandi, Serge MA Somda, John J Reilly

**Affiliations:** a Laboratoire de Nutrition, Département de Biologie Animale, Faculté des Sciences et Techniques, Université Cheikh Anta Diop de Dakar, BP 5005 Dakar Fann, Senegal.; b Nutrition Research Centre, Ghana Atomic Energy Commission, Accra, Ghana.; c Association Tunisienne des Sciences de la Nutrition, Tunis, Tunisia.; d Unité Mixte de Recherche Nutrition et Alimentation CNESTEN-Université Ibn Tofail, Rabat, Morocco.; e Biochemistry Department; Victoria Hospital; Ministry of Health and Quality of Life, Quatre Bornes, Mauritius.; f International Atomic Energy Agency, Vienna International Centre, Vienna, Austria.; g Department of Epidemiology and Biostatistics, School of Public Health and Social Sciences, Dar el Salaam, United Republic of Tanzania.; h Department of Food, Nutrition and Dietetics, Kenyatta University, Nairobi, Kenya.; i Physical Activity, Sport and Recreation, Faculty of Health Sciences, North-West University, Potchefstroom, South Africa.; j Ministry of Health and Social Services, Windhoek, Namibia.; k Centre Muraz, Bobo-Dioulasso, Burkina Faso.; l School of Psychological Science and Health, University of Strathclyde, Glasgow, Scotland.

## Abstract

**Objective:**

To compare the World Health Organization (WHO) body mass index (BMI)-for-age definition of obesity against measured body fatness in African children.

**Methods:**

In a prospective multicentre study over 2013 to 2017, we recruited 1516 participants aged 8 to 11 years old from urban areas of eight countries (Ghana, Kenya, Mauritius, Morocco, Namibia, Senegal, Tunisia and United Republic of Tanzania). We measured height and weight and calculated BMI-for-age using WHO standards. We measured body fatness using the deuterium dilution method and defined excessive body fat percentage as > 25% in boys and > 30% in girls. We calculated the sensitivity and specificity of BMI z-score > +2.00 standard deviations (SD) and used receiver operating characteristic analysis and the Youden index to determine the optimal BMI z-score cut-off for classifying excessive fatness.

**Findings:**

The prevalence of excessive fatness was over three times higher than BMI-for-age-defined obesity: 29.1% (95% CI: 26.8 to 31.4; 441 children) versus 8.8% (95% CI: 7.5 to 10.4; 134 children). The sensitivity of BMI z-score > +2.00 SD was low (29.7%, 95% CI: 25.5 to 34.2) and specificity was high (99.7%, 95% CI: 99.2 to 99.9). The receiver operating characteristic analysis found that a BMI z-score +0.58 SD would optimize sensitivity, and at this cut-off the area under the curve was 0.86, sensitivity 71.9% (95% CI: 67.4 to 76.0) and specificity 91.1% (95% CI: 89.2 to 92.7).

**Conclusion:**

While BMI remains a practical tool for obesity surveillance, it underestimates excessive fatness and this should be considered when planning future African responses to the childhood obesity pandemic.

## Résumé

**Objectif:**

Comparer la définition de l'obésité de l'Organisation mondiale de la Santé basée sur l'indice de masse corporelle (IMC) selon l'âge à la masse grasse mesurée chez les enfants africains.

**Méthodes:**

Dans le cadre d'une étude prospective multicentrique menée entre 2013 et 2017, nous avons recruté 1516 participants âgés de 8 à 11 ans dans des zones urbaines situées dans huit pays (Ghana, Kenya, Maroc, Maurice, Namibie, République-Unie de Tanzanie, Sénégal et Tunisie). Nous avons mesuré leur taille et leur poids et calculé leur IMC par rapport à leur âge en utilisant les normes de l'OMS. Nous avons mesuré la masse grasse à l'aide de la méthode de dilution du deutérium, et défini le taux de masse grasse excessive comme étant > 25% pour les garçons et > 30% pour les filles. Nous avons calculé la sensibilité et la spécificité du Z-score de l'IMC > +2,00 écarts types (ET) et utilisé une analyse de la fonction d'efficacité du récepteur et l’indice de Youden afin de déterminer la valeur limite optimale du Z-score de l'IMC pour classifier la masse grasse excessive.

**Résultats:**

La prévalence de la masse grasse excessive était plus de trois fois supérieure à la prévalence de l'obésité définie en fonction de l'IMC selon l'âge: 29,1% (IC à 95%: 26,8-31,4; 441 enfants) contre 8,8% (IC à 95%: 7,5-10,4; 134 enfants). La sensibilité du Z-score de l'IMC > +2,00 ET était faible (29,7%, IC à 95%: 25,5-34,2), tandis que la spécificité était élevée (99,7%, IC à 95%: 99,2-99,9). L'analyse de la fonction d'efficacité du récepteur a révélé qu'un Z-score de l'IMC de +0,58 ED optimiserait la sensibilité, et qu'à cette valeur limite, l'aire sous la courbe était de 0,86, la sensibilité de 71,9% (IC à 95%: 67,4-76,0) et la spécificité de 91,1% (IC à 95%: 89,2-92,7).

**Conclusion:**

Si l'IMC reste est un outil pratique pour surveiller l'obésité, il sous-évalue la masse grasse excessive. Cela doit être pris en compte lors de la planification des futures mesures africaines de lutte contre la pandémie d'obésité chez l'enfant.

## Resumen

**Objetivo:**

Comparar la definición de obesidad por edad del índice de masa corporal (IMC) de la Organización Mundial de la Salud (OMS) con la grasa corporal medida en niños africanos.

**Métodos:**

En un estudio prospectivo multicéntrico realizado entre 2013 y 2017, se reclutaron 1516 participantes de edades comprendidas entre los 8 y los 11 años de zonas urbanas de ocho países (Ghana, Kenya, Marruecos, Mauricio, Namibia, República Unida de Tanzania, Senegal y Túnez). Se midieron la altura y el peso y calculamos el IMC por edad utilizando los estándares de la OMS. Se midió la grasa corporal mediante el método de dilución de deuterio y se definió el porcentaje de grasa corporal excesiva como > 25 % en los niños y > 30 % en las niñas. Se calculó la sensibilidad y especificidad del IMC con desviaciones estándar (DE) de los valores Z de > +2,00 y se utilizó el análisis de las características operativas del receptor y el índice Youden para determinar el límite óptimo del IMC z-score para clasificar el exceso de grasa.

**Resultados:**

La prevalencia de la obesidad excesiva fue más de tres veces superior a la obesidad definida por el IMC por edad: 29,1 % (IC del 95 %: 26,8 a 31,4; 441 niños) en comparación con un 8,8 % (IC del 95 %: 7,5 a 10,4; 134 niños). La sensibilidad del IMC con DE de los valores Z > +2,00 fue baja (29,7 %, IC del 95 %: 25,5 a 34,2) y la especificidad fue alta (99,7 %, IC del 95 %: 99,2 a 99,9). El análisis de las características operativas del receptor encontró que un IMC z-score +0,58 DE optimizaría la sensibilidad, y en este corte el área bajo la curva era de 0,86, con una sensibilidad del 71,9 % (IC del 95 %: 67,4 a 76,0) y una especificidad del 91,1 % (IC del 95 %: 89,2 a 92,7).

**Conclusión:**

Aunque el IMC sigue siendo una herramienta práctica para la monitorización de la obesidad, subestima el exceso de grasa y esto debería tenerse en cuenta a la hora de planificar las futuras respuestas africanas a la pandemia de obesidad infantil.

## ملخص

**الغرض:**

مقارنة بين السمنة المقيسة في الأطفال الأفارقة، وتعريف البدانة القائم على مؤشر كتلة الجسم (BMI) بالنسبة للسن، والتابع لمنظمة الصحة العالمية.

**الطريقة:**

في دراسة استباقية متعددة المراكز خلال الفترة من 2013 إلى 2017، قمنا بتسجيل 1516 مشاركًا تتراوح أعمارهم بين 8 إلى 11 عامًا من المناطق الحضرية في ثماني بلدان (السنغال، والمغرب، وتونس، وجمهورية تنزانيا المتحدة، وغانا، وكينيا، وموريشيوس، وناميبيا).قمنا بقياس الطول والوزن وحساب مؤشر كتلة الجسم (BMI) بالنسبة للسن، باستخدام معايير منظمة الصحة العالمية.قمنا بقياس سمنة الجسم باستخدام طريقة تخفيف الديوتيريوم، وحددنا النسبة المئوية للدهون الزائدة في الجسم لتكون أكثر من 25٪ في الأولاد، وأكثر من 30٪ في البنات.قمنا بحساب حساسية وخصوصية المحصلة المعيارية لمؤشر كتلة الجسم لتكن أكبر من +2.00 بالنسبة للانحرافات المعيارية (SD)، واستعنا بتحليل خصائص التشغيل للمستقبٍل ومؤشر يودنلتحديد الحد الأمثل للمحصلة المعيارية لمؤشر كتلة الجسم، بهدف تصنيف السمنة المفرطة.

**النتائج:**

كان انتشار السمنة المفرطة أعلى أكثر من ثلاث مرات من السمنة التي يحددها مؤشر كتلة الجسم بالنسبة للسن: 29.1٪ (فاصل الثقة 95٪: 26.8 إلى 31.4؛ 441 طفلاً) مقابل 8.8٪ (فاصل الثقة 95٪: 7.5 إلى 10.4، 134 طفلاً).كانت حساسية الانحراف المعياري (SD) - الأكبر من +2.00 بالنسبة للمحصلة المعيارية لمؤشر كتلة الجسم- منخفضة، (29.7٪، بفاصل ثقة 95٪: 25.5 إلى 34.2) وكانت الخصوصية عالية (99.7٪، بفاصل ثقة 95٪: 99.2 إلى 99.9).اكتشف تحليل خصائص التشغيل للمستقبٍل أن الانحراف المعياري بنسبة +0.58 - للمحصلة المعيارية لمؤشر كتلة الجسم- سوف يُحسن من الحساسية، وأنه عند هذا الحد كانت المساحة تحت المنحنى 0.86، وكانت الحساسية 71.9٪ (فاصل ثقة 95٪: 67.4 إلى 76.0)، وخصوصية 91.1٪ (فاصل ثقة 95٪: 89.2 إلى 92.7).

**الاستنتاج:**

بينما يظل مؤشر كتلة الجسم أداة عملية لمراقبة السمنة، إلا أنه يقلل من السمنة المفرطة، ويجب وضع ذلك في الاعتبار عند التخطيط لاستجابات أفريقية مستقبلية لوباء البدانة في مرحلة الطفولة.

## 摘要

**目的:**

将世卫组织 (WHO) 年龄别身体质量指数 (BMI-for-age) 对肥胖的定义与所测量的非洲儿童体脂率进行对比。

**方法:**

在 2013 年至 2017 年的一项前瞻性、多中心的研究中，我们从 8 个国家的城市地区（加纳，肯尼亚，摩洛哥，毛里求斯，纳米比亚，塞内加尔，突尼斯和坦桑尼亚联合共和国）招募了 1516 名 8 至 11 岁的参与者。我们使用 WHO 标准测量了参与者的身高和体重，并计算了年龄别身体质量指数。我们使用重水同位素法测量了参与者的体脂，并定义过度肥胖百分位数为男孩 >25％，女孩 >30％。我们计算了 BMI z 评分 >+2.00 标准差 (SD) 的敏感性和特异性，并使用受试者工作特征分析和约登指数来确定过度肥胖分类的最佳 BMI z 评分的界限值。

**结果:**

过度肥胖的患病率比年龄别身体质量指数所定义的肥胖高出三倍以上：29.1％（95％ 置信区间，CI：26.8 至 31.4；441 名儿童）与 8.8％（95％ 置信区间，CI：7.5 至 10.4；134 名儿童）。BMI z 评分 >+2.00 标准差 (SD) 的敏感性低（29.7％，95％ 置信区间，CI：25.5 至 34.2），特异性高（99.7％，95％ 置信区间，CI：99.2 至 99.9）。受试者工作特征分析发现 BMI z 评分 +0.58 标准差 (SD) 将优化敏感性，并在此界限值时，曲线下区域为 0.86，敏感性为 71.9％（95％ 置信区间，CI：67.4 至 76.0），特异性为 91.1％（95％ 置信区间，CI：89.2 至 92.7）。

**结论:**

虽然身体质量指数 (BMI) 仍然是肥胖监测的实用工具，但它低估了过度肥胖的患病率，因此，在规划未来非洲应对儿童肥胖率快速上升的情况时应慎重考虑。

## Резюме

**Цель:**

Сравнение определения ожирения, принятого Всемирной организацией здравоохранения (ВОЗ) на основании значения «индекс массы тела (ИМТ)-возраст», с измеренными величинами упитанности африканских детей.

**Методы:**

В перспективном многоцентровом исследовании в период с 2013 по 2017 год приняли участие 1516 участников в возрасте от 8 до 11 лет из городских районов восьми стран (Гана, Кения, Маврикий, Марокко, Намибия, Объединенная Республика Танзания, Сенегал и Тунис). Детей взвешивали и измеряли их рост, после чего вычисляли показатель «ИМТ-возраст» согласно стандартам ВОЗ. Также измерялось содержание жировой ткани в организме методом дейтериевого разбавления; содержание жировой ткани считалось избыточным, если оно превышало 25% у мальчиков и 30% у девочек. Авторы вычислили чувствительность и специфичность z-оценки ИМТ >+ 2,00 стандартного отклонения (СО) и воспользовались методом анализа характеристических показателей правильности обнаружения сигналов индексом Юдена для определения оптимального порога z-оценки по ИМТ в вопросе классификации наличия избытка жировой ткани.

**Результаты:**

Распространенность избытка жировой ткани более чем в три раза превышала частоту ожирения, определяемую показателем «ИМТ-возраст»: 29,1% (95%-й ДИ: 26,8–31,4; 441 ребенок) в сравнении с 8,8% (95%-й ДИ: 7,5–10,4; 134 ребенка). Чувствительность z-оценки ИМТ >+ 2,00 СО была низкой (29,7%, 95%-й ДИ: 25,5–34,2), а специфичность — высокой (99,7%, 95%-й ДИ: 99,2–99,9). Анализ характеристических особенностей правильности обнаружения сигналов позволил обнаружить, что оптимизация чувствительности возможна при использовании z-оценки ИМТ + 0,58 СО и что при этом пороговом значении площадь под кривой составляла 0,86, чувствительность — 71,9% (95%-й ДИ: 67,4–76,0), а специфичность — 91,1% (95%-й ДИ: 89,2–92,7).****

**Вывод:**

Несмотря на то что измерение ИМТ остается практическим средством выявления ожирения, оно недооценивает содержание избыточной жировой ткани в организме, и это следует учитывать при планировании мероприятий по борьбе с пандемией детского ожирения в Африке.

## Introduction

Childhood obesity is now a pandemic, heralding a substantial burden of future noncommunicable diseases,^1^^,^^2^ despite the established burden of underweight in low- and middle-income countries. Changes in diet and reduced physical activity among adolescent boys and girls^3^ have occurred across Africa, currently most evident in urban areas, although rural areas are also affected.^4^ The World Health Organization (WHO) *Report of the Commission on Ending Childhood Obesity*^1^ advocated more surveillance of the prevalence of obesity to plan where and when to intervene, and to measure the effectiveness of future interventions.^1^

Body mass index (BMI)-for-age is a well-established indicator for surveillance of paediatric obesity. The WHO child growth standards define obesity in school-aged children as BMI z-score > +2.00 standard deviations (SD).^5^ Systematic reviews have shown that, as in adults,^6^ high BMI-for-age identifies children with the highest body fatness and the highest risk of co-morbidities. However, the indicator is conservative as it fails to identify children who are excessively fat, but who do not have high BMI-for-age.^7^^–^^10^ There are several problems with this evidence. First, few studies tested the diagnostic performance of the WHO BMI-for-age definition of obesity, focusing on definitions based on national BMI reference data or the International Obesity Task Force definition.^9^^,^^10^ Second, few studies assessed the diagnostic performance of BMI-for-age against a measure of body fatness with low bias and acceptable individual diagnostic accuracy such as total body water.^11^^,^^12^ Finally, the applicability of the evidence to African children is unclear; bias in the estimation of excessive body fatness by BMI varies across populations in adults.^13^ The extent to which such bias is population-specific for children too is less clear, although compared with Europeans, South-East Asian children have higher body fatness than would be expected from their BMI.^14^

The aim of this study was to compare the prevalence of the WHO BMI-for-age definition of obesity against the prevalence of excessive body fatness in a relatively large sample of African children.

## Methods

### Study design

This design for this prospective, multicentre, data-pooling study, was agreed at the first meeting of the Reducing Obesity Using Nuclear Techniques To Design Interventions study in 2012. We followed the Standards for Reporting of Diagnostic Accuracy Studies^15^ for the conduct and reporting of the study. Sampling and study procedures originally took place across 11 African centres between 2013 and 2017. We aimed to recruit around 150 participants per country (a larger sample was used in the United Republic of Tanzania, because some of the study aims there required a larger sample). As the nutrition and physical activity transitions in Africa have disproportionately affected urban children,^3^^,^^4^ we focused the sampling in urban areas. In each country, we used a multistage random sampling method to select at least four to five urban public schools in one district or state, followed by school sampling frames of all classes corresponding to the target age group and sex. More details of the methods are available from the corresponding author.

Children meeting the inclusion criteria were recruited to participate in the study after submission of a signed informed consent form by a parent. Data collection was conducted during the school year. Ethical approval was obtained from local research boards or committees in each country. Participants were eligible for inclusion if they were age 8 to 11 years and provided consent or assent for participation; they were excluded if they were outside the study age range, had ill health that would have precluded participation or were not present in school after two consecutive visits.

### Anthropometric measures

The study used a common protocol and standard operating procedures across all countries. Before data collection started all researchers were trained in data collection methods by a team of experienced researchers and fieldworkers during a 1-week residential course in South Africa. The height of children was measured to the nearest 0.1 cm using a Seca stadiometer, and weight to 0.1 kg in light indoor clothing using a Seca scale (Seca, London, England). From the height and weight measures, we calculated BMI for each child as weight divided by height squared (kg/m^2^) and then computed age- and sex-specific z-score relative to the WHO BMI-for-age reference^5^ using the Stata zanthro package (Stata Corp., College Station, United States of America). We defined obesity as BMI z-score > +2.00 SD and overweight (including obesity) as BMI z-score > +1.00 SD

### Body water measures

We aimed to measure total body water in all participants using the deuterium dilution method, as described previously.^11^^,^^14^ We used standard operating procedures, with training support provided for all countries via a combination of residential and on-site training by experts recruited by the International Atomic Energy Agency. Ideally, body fatness measurement methods are multicomponent, based on measures of total body water plus body density or total body mineral. However, such methods are laboratory-based and impractical for large-scale epidemiological studies. While not a criterion method, body fatness measured by total body water is practical for large epidemiological studies and provides accurate measures of fatness which are unbiased relative to multicomponent methods.^11^^,^^12^

The total body water measures were made on the same day as the height, weight and waist circumference measures. Accurate measurement of total body water requires a normal hydration status. We therefore asked participants and their families to have normal fluid and food intake on the day before the estimation of total body water and to avoid vigorous exercise after the final meal of the previous day to avoid dehydration and depletion of glycogen stores. Deuterium oxide-labelled water (99.8% purity; Cambridge Isotope Laboratories Inc., Andover, USA) accurately weighed (0.001 g precision) was orally administered to the children according to their body weight (0.5 g deuterium oxide per kg) followed by 50 mL of local tap water. Children were asked not to eat or drink for at least 30 minutes before receiving the deuterium-labelled water and to void their bladders before dosing. Baseline (pre-dose) saliva samples were collected from each participant by rotating a cotton-wool ball in the buccal cavity of the mouth until well soaked. Saliva was collected into a clean sterile and dry tube using a 20 mL disposable syringe. Participants were then requested to drink the labelled water dose under supervision and two further saliva samples were collected at 3 hours and 4 hours after the dose using the method described above. All saliva samples were stored at 4 °C until their arrival to the laboratory for storage at −20 °C until analysis. Analysis was carried out with Fourier transform infrared spectroscopy (FTIR 8400S spectrophotometer, Shimadzu Kyoto, Japan) in accordance with International Atomic Energy Agency protocols.^16^

We converted measures of total body water to total body fat using established age- and sex-specific constants for the hydration of fat-free mass,^16^^,^^17^ as described elsewhere.^14^ Quality control procedures, with four stringent criteria described in detail elsewhere,^16^ were applied to the measures of enrichment of deuterium required for the total body water measures and to the estimates of total body water, total body fat and body fat percentage. These quality control measures were: (i) deuterium enrichment of each of the two post-dose samples should be within 2% of the mean of the two post-dose samples; (ii) measured enrichment should lie within an expected range of normal enrichments based on the body weight of the child (outliers in the total body-water-to-height relationship were identified and excluded); (iii) outliers in body fat percentage were identified and excluded (e.g. large mismatches between body fat percentage and BMI z-score or unphysiological body fat percentage measures);^16^ and (iv) if more than 10% of total body water measures from any centre failed to meet the quality control criteria, then we excluded all data from that centre from the pooled analyses. Based on these criteria, we excluded data from three out of 11 original participating countries (Benin, Mali and Uganda), so that the present study is based on data from eight countries (Ghana, Kenya, Mauritius, Morocco, Namibia, Senegal, Tunisia and United Republic of Tanzania) and 1516 children. Among these, 2% of total body water measures were rejected for quality control reasons and were not included in the analyses reported here.

We expressed total body fatness as a percentage of body weight. Many studies have established that a high body fatness, even in childhood, has a range of adverse health consequences, with most focusing on the cardiometabolic consequences, as summarized by systematic reviews.^7^^,^^8^ One report on the relationship between body fatness and cardio-metabolic risk in childhood used a skinfold thickness method previously validated against a multicomponent model to measure body fatness.^18^ The researchers found a marked increase in cardiometabolic risk profile at body fat > 25% in boys and > 30% in girls, across a wide age range. We therefore used this definition of excessive fatness (true positive in the receiver operator characteristic analysis) in the present study. As in previous studies,^9^^,^^10^ the conclusions were not greatly affected by the definition of excessive fatness (data are available from the corresponding author).

### Data management

Training in data management, data sharing and data quality control was provided during a 1-week data management residential training course in Benin in 2014. Throughout the study, support was provided by site visits and online chat or email by a central data management coordinator from Burkina Faso recruited by the International Atomic Energy Agency. The weight and height measures, BMI-for-age z-scores and total body water-derived measures of body fatness were all made prospectively and independently and the results of each measure were not available at the time of the other measures.

### Analysis

We used standard diagnostic performance indicators to determine the extent to which BMI z-score > +2.00 SD identified children with excessive fatness. We calculated sensitivity (proportion of real positive values among all the recorded positive values), specificity (proportion of real negative values among all the negative values), and positive and negative predictive values for the total sample. We used the Youden index method to determine the optimal BMI z-score cut-off for optimizing the sensitivity and specificity for identifying excessive fatness. We used Spearman rank-order correlation to test the association between countries’ total BMI-for-age z-score and total body fat percentage. We also made an exploratory analysis of possible geographical differences in the results by grouping the countries into three geographically defined sub-groups: sub-Saharan Africa (Ghana, Kenya, Namibia, Senegal and United Republic of Tanzania), North Africa (Morocco and Tunisia) and an African island (Mauritius).

## Results

**Fig. 1:**
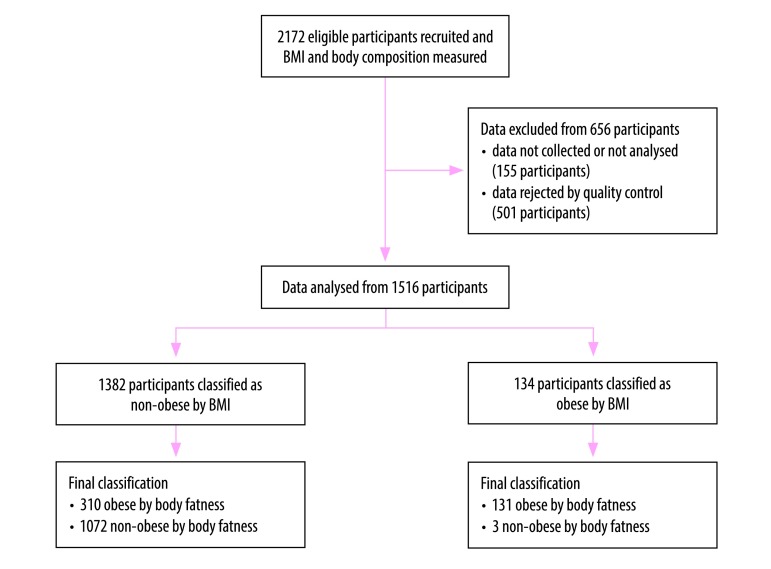
Flowchart on the inclusion of participants to compare methods to measure overweight in children in eight African countries, 2013–2017

**Fig. 2:**
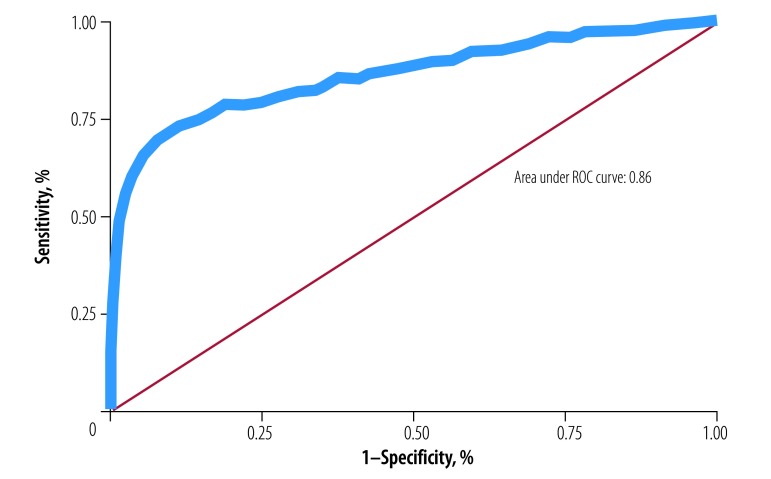
Relationships between body mass index-for-age z-score and body fat percentage among children in eight African countries, by geographical area, 2013–2017

Fig. 1 shows the flowchart of the study. Of the 2172 children recruited to the study, eligible data were available from 1516 (69.8%). The age and anthropometric characteristics of the eligible participants are shown in Table 1. The mean age was 9.6 years (95% confidence interval, CI: 9.5 to 9.7) and median age was 10 years (interquartile range, IQR: 9 to 11). The median BMI-for-age z-score was −0.35 (IQR: −1.09 to 0.71) and median body fat percentage was 22.65% (IQR: 17.43 to 29.60). Fig. 2 provides more detail on the distribution of body fatness and BMI-for-age z-scores. The prevalence of excessive fatness was 29.1% (95% CI: 26.8 to 31.4; 441 children). Overall, the prevalence of obesity by the WHO BMI-for-age criterion was 8.8% (95% CI: 7.5 to 10.4; 134 children) (Table 2) and of overweight was 19.5% (95% CI: 17.6 to 21.6; 296 children) (Table 3).

BMI: body mass index.

Notes: Children were originally recruited and assessed for BMI and body composition in 11 countries. Data were rejected for quality control reasons from Benin, Mali and Uganda. The final analysis was therefore based on data from eight countries: Ghana, Kenya, Mauritius, Morocco, Namibia, Senegal, Tunisia and United Republic of Tanzania.

**Table 1:** Age and anthropometric characteristics of participants in the study of body mass index-for-age and body fatness among children in eight African countries, 2013–2017

Sex, by country	No. (%) of children^a^	Median (IQR)				
		Age, years	BMI z-score	Body fat percentage	Fat mass index, kg/m^2^	Fat-free mass index, kg/m^2^
**Ghana**						
Boys	71 (37.4)	10 (9–11)	−0.87 (−1.17 to −0.21)	15.92 (12.57 to 19.94)	2.37 (1.98 to 3.24)	12.98 (12.08 to 13.75)
Girls	119 (62.6)	10 (9–10)	−0.66 (−1.16 to 0.09)	18.72 (15.65 to 22.62)	2.85 (2.33 to 3.74)	12.63 (11.77 to 13.38)
Total	190 (100.0)	10 (9–11)	−0.70 (−1.16 to −0.05)	18.03 (14.40 to 21.08)	2.65 (2.14 to 3.42)	12.77 (11.95 to 13.70)
**Kenya**						
Boys	84 (46.9)	10 (9–11)	−0.91 (−1.34 to −0.30)	22.94 (17.93 to 28.61)	3.58 (2.68 to 5.01)	12.29 (10.92 to 13.64)
Girls	95 (53.1)	10 (9–11)	−0.69 (−1.36 to −0.05)	24.14 (19.43 to 27.49)	3.71 (2.85 to 4.86)	12.47 (10.73 to 14.53)
Total	179 (100.0)	10 (9–11)	−0.82 (−1.35 to −0.15)	23.57 (19.34 to 28.11)	3.64 (2.75 to 4.86)	12.34 (10.84 to 13.90)
**Mauritius**						
Boys	82 (53.6)	10 (9–11)	0.76 (−1.01 to 1.86)	25.28 (18.67 to 33.23)	4.12 (2.75 to 7.36)	13.14 (11.74 to 14.67)
Girls	71 (46.4)	10 (9–11)	0.56 (−0.55 to 1.84)	32.11 (24.62 to 37.66)	5.67 (3.96 to 8.39)	12.64 (11.44 to 14.68)
Total	153 (100.0)	10 (9–11)	0.68 (−0.76 to 1.84)	28.80 (21.65 to 35.48)	4.96 (3.41 to 7.71)	13.01 (11.64 to 14.68)
**Morocco**						
Boys	94 (50.3)	9 (8–10)	−0.24 (−1.00 to 0.51)	19.76 (16.31 to 24.57)	3.08 (2.49 to 4.17)	12.76 (12.15 to 13.66)
Girls	93 (49.7)	9 (8–10)	−0.33 (−0.99 to 0.42)	25.69 (21.91 to 30.11)	4.07 (3.20 to 5.07)	11.97 (11.06 to 12.62)
Total	187 (100.0)	9 (8–10)	−0.27 (−0.99 to 0.51)	23.23 (18.30 to 28.60)	3.70 (2.76 to 4.72)	12.36 (11.63 to 13.27)
**Namibia**						
Boys	66 (43.7)	10 (9–11)	−0.08 (−0.91 to 1.09)	22.84 (18.85 to 30.76)	3.60 (2.70 to 5.73)	12.92 (12.06 to 13.69)
Girls	85 (56.3)	10 (9–11)	0.42 (−0.76 to 1.64)	32.69 (26.76 to 39.06)	5.38 (4.14 to 8.71)	11.94 (11.01 to 13.18)
Total	151 (100.0)	10 (9–11)	0.19 (−0.84 to 1.44)	27.97 (22.32 to 37.50)	4.70 (3.38 to 7.42)	12.59 (11.44 to 13.41)
**Senegal**						
Boys	70 (47.9)	10 (9–11)	−1.29 (−1.84 to −0.71)	13.43 (10.64 to 19.99)	1.95 (1.49 to 3.07)	12.50 (11.72 to 13.10)
Girls	76 (52.1)	10 (9–10)	−1.40 (−2.15 to −0.58)	19.30 (15.80 to 24.91)	2.62 (2.13 to 3.52)	11.41 (10.71 to 12.04)
Total	146 (100.0)	10 (9–10)	−1.32 (−2.05 to −0.60)	16.70 (12.76 to 22.61)	2.35 (1.79 to 3.34)	11.84 (11.12 to 12.66)
**Tunisia**						
Boys	80 (51.0)	9 (9–10)	0.04 (−0.65 to 0.99)	23.49 (20.30 to 26.86)	3.86 (3.19 to 5.00)	12.56 (11.89 to 13.75)
Girls	77 (49.0)	10 (8–10)	0.31 (−0.65 to 1.18)	30.03 (25.57 to 33.89)	4.89 (3.99 to 6.34)	11.94 (11.13 to 12.82)
Total	157 (100.0)	9 (8–10)	0.10 (−0.65 to 1.12)	26.03 (22.88 to 31.37)	4.29 (3.54 to 5.77)	12.37 (11.60 to 13.29)
**United Republic of Tanzania**						
Boys	158 (44.8)	10 (9–11)	0.04 (−0.58 to 1.12)	18.50 (15.10 to 24.90)	3.00 (2.41 to 4.83)	13.42 (12.73 to 14.34)
Girls	195 (55.2)	10 (9–11)	0.02 (−0.80 to 0.92)	23.30 (19.30 to 31.10)	3.73 (2.93 to 5.62)	12.70 (11.89 to 13.63)
Total	353 (100.0)	10 (9–11)	0.02 (−0.67 to 0.95)	21.50 (17.00 to 29.40)	3.43 (2.60 to 5.37)	13.00 (12.27 to 14.14)
**Total**						
Boys	705 (46.5)	10 (9–11)	−0.37 (−1.09 to 0.69)	20.47 (15.60 to 26.09)	3.25 (2.40 to 4.57)	12.92 (12.06 to 13.90)
Girls	811 (53.5)	10 (9–10)	−0.33 (−1.09 to 0.72)	24.90 (19.37 to 31.46)	3.91 (2.87 to 5.64)	12.23 (11.31 to 13.31)
Total	1516 (100.0)	10 (9–11)	−0.35 (−1.09 to 0.71)	22.65 (17.43 to 29.60)	3.59 (2.60 to 5.17)	12.59 (11.64 to 13.63)

BMI: body mass index; IQR: interquartile range.

^a^ Number of records removed from original samples: Ghana (4), Kenya (1), Mauritius (3), Morocco (3), Namibia (4), Senegal (10), Tunisia (2) and United Republic of Tanzania (3).

Notes: BMI was calculated as weight in kg divided by height in m^2^ and z-scores were obtained from the World Health Organization BMI-for-age child growth standards.^5^ Body fat percentage was measured using deuterium oxide dilution. Fat mass index was calculated as fat mass in kg divided by height in m^2^, with fat mass measured from total body water. Fat free mass index was calculated as fat free mass in kg divided by height in m^2^, with fat-free mass measured from total body water. Measures were made in 2014–2017 in Kenya and United Republic of Tanzania and in 2013–2015 in all other countries.

BMI: body mass index.

Note: Areas were defined as follows: sub-Saharan Africa (Ghana, Kenya, Namibia, Senegal and United Republic of Tanzania), North Africa (Morocco and Tunisia) and African island (Mauritius).

**Table 2:** Comparison of obesity defined by body mass index-for-age and by body fatness among children in eight African countries, by geographical area, 2013–2017

Obesity defined by BMI-for-age^a^	Obesity defined by body fatness,^b^ no. (%) of children		
	No	Yes	Total
**Sub-Saharan Africa**			
Ghana			
No	183 (100.0)	5 (71.4)	188 (99.0)
Yes	0 (0.0)	2 (28.6)	2 (1.0)
Total	183 (100.0)	7 (100.0)	190 (100.0)
Kenya			
No	125 (100.0)	50 (92.6)	175 (97.8)
Yes	0 (0.0)	4 (7.4)	4 (2.2)
Total	125 (100.0)	54 (100.0)	179 (100.0)
Namibia			
No	75 (100.0)	49 (64.5)	124 (82.1)
Yes	0 (0.0)	27 (35.5)	27 (17.9)
Total	75 (100.0)	76 (100.0)	151 (100.0)
Senegal			
No	130 (100.0)	12 (75.0)	142 (97.3)
Yes	0 (0.0)	4 (25.0)	4 (2.7)
Total	130 (100.0)	16 (100.0)	146 (100.0)
United Republic of Tanzania			
No	259 (98.9)	48 (52.7)	307 (87.0)
Yes	3 (1.1)	43 (47.3)	46 (13.0)
Total	262 (100.0)	91 (100.0)	353 (100.0)
All			
No	772 (99.6)	164 (67.2)	936 (91.9)
Yes	3 (0.4)	80 (32.8)	83 (8.1)
Total	775 (100.0)	244 (100.0)	1019 (100.0)
**North Africa**			
Morocco			
No	141 (100.0)	37 (80.4)	178 (95.2)
Yes	0 (0.0)	9 (19.6)	9 (4.8)
Total	141 (100.0)	46 (100.0)	187 (100.0)
Tunisia			
No	89 (100.0)	60 (88.2)	149 (94.9)
Yes	0 (0.0)	8 (11.8)	8 (5.1)
Total	89 (100.0)	68 (100.0)	157 (100.0)
All			
No	230 (100.0)	97 (85.1)	327 (95.1)
Yes	0 (0.0)	17 (14.9)	17 (4.9)
Total	230 (100.0)	114 (100.0)	344 (100.0)
**African island**			
Mauritius			
No	70 (100.0)	49 (59.0)	119 (77.8)
Yes	0 (0.0)	34 (41.0)	34 (22.2)
Total	70 (100.0)	83 (100.0)	153 (100.0)
**All countries**			
No	1072 (99.7)	310 (70.3)	1382 (91.2)
Yes	3 (0.3)	131 (29.7)	134 (8.8)
Total	1075 (100.0)	441 (100.0)	1516 (100.0)

BMI: body mass index.

^a^ We measured height and weight and calculated obesity from BMI-for-age using the World Health Organization reference z-score > +2.00 standard deviations.

^b^ We measured body fatness using the deuterium dilution method and defined excessive body fat percentage as > 25% in boys and > 30% in girls.^11^^,^^12^

**Table 3:** Comparison of overweight defined by body mass index-for-age and obesity defined by body fatness among children in eight African countries, by geographical area, 2013–2017

Overweight defined by BMI-for-age^a^	Obesity defined by body fatness,^b^ no. (%) of children		
	No	Yes	Total
**Sub-Saharan Africa**			
Ghana			
No	182 (99.5)	2 (28.6)	184 (96.8)
Yes	1 (0.5)	5 (71.4)	6 (3.2)
Total	183 (100.0)	7 (100.0)	190 (100.0)
Kenya			
No	122 (97.6)	48 (88.9)	170 (95.0)
Yes	3 (2.4)	6 (11.1)	9 (5.0)
Total	125 (100.0)	54 (100.0)	179 (100.0)
Namibia			
No	75 (100.0)	26 (34.2)	101 (66.9)
Yes	0 (0.0)	50 (65.8)	50 (33.1)
Total	75 (100.0)	76 (100.0)	151 (100.0)
Senegal			
No	130 (100.0)	7 (43.8)	137 (93.8)
Yes	0 (0.0)	9 (56.2)	9 (9.2)
Total	130 (100.0)	16 (100.0)	146 (100.0)
United Republic of Tanzania			
No	246 (93.9)	21 (23.1)	267 (75.6)
Yes	16 (6.1)	70 (76.9)	86 (24.4)
Total	262 (100.0)	91 (100.0)	353 (100.0)
All			
No	755 (97.4)	104 (42.6)	859 (84.3)
Yes	20 (2.6)	140 (57.4)	160 (15.7)
Total	775 (100.0)	244 (100.0)	1019 (100.0)
**North Africa**			
Morocco			
No	137 (97.2)	21 (45.7)	158 (84.5)
Yes	4 (2.8)	25 (54.3)	29 (15.5)
Total	141 (100.0)	46 (100.0)	187 (100.0)
Tunisia			
No	84 (94.4)	29 (42.6)	113 (72.0)
Yes	5 (5.6)	39 (57.4)	44 (28.0)
Total	89 (100.0)	68 (100.0)	157 (100.0)
All			
No	221 (96.1)	50 (43.9)	271 (78.8)
Yes	9 (3.9)	64 (56.1)	73 (21.2)
Total	230 (100.0)	114 (100.0)	344 (100.0)
**African island**			
Mauritius			
No	64 (91.4)	26 (31.3)	90 (58.8)
Yes	6 (8.6)	57 (68.7)	63 (41.2)
Total	70 (100.0)	83 (100.0)	153 (100.0)
**All countries**			
No	1040 (96.7)	180 (40.8)	1220 (80.5)
Yes	35 (3.3)	261 (59.2)	296 (19.5)
Total	1075 (100.0)	441 (100.0)	1516 (100.0)

BMI: body mass index.

^a^ We measured height and weight and calculated overweight from BMI-for-age using the World Health Organization reference z-score > +1.00 standard deviations.^5^

^b^ We measured body fatness using the deuterium dilution method and defined excessive body fat percentage as > 25% in boys and > 30% in girls.^11^^,^^12^

In the whole sample, the sensitivity of BMI z-score > +2.00 SD for identifying excessively fat children was 29.7% (95% CI: 25.5 to 34.2), specificity was 99.7% (95% CI: 99.2 to 99.9), positive predictive value 97.8% (95% CI: 93.6 to 99.5) and negative predictive value 77.6% (95% CI: 75.3 to 79.7). The sensitivity of BMI z-score > +2.00 SD to identify excessively fat children varied little between boys and girls (66/203 for boys versus 65/238 for girls). In the whole sample BMI z-score > 1.00 SD had sensitivity of 59.2% (95% CI: 54.4 to 63.8) and specificity of 96.7% (95% CI: 95.5 to 97.7; Table 4). Analysis of the data by country and in the three population sub-groups is shown in Table 2 and Table 3. Sensitivity was lower in the North African and Island populations than the sub-Saharan Africans. The rank order correlation between country median BMI z-score and country fat mass index was high (*r* = 0.6).

**Table 4:** Comparison of World Health Organization body mass index-for-age cut-offs for obesity and overweight and the empirically determined optimal cut-off for identifying excessive fatness among children in eight African countries, 2013–2017

Diagnostic performance measure^a^	BMI z-score > +2.00 SD				BMI z-score > +1.00 SD				BMI z-score +0.58 SD^b^		
	No. of children	Total no.	% (95% CI)		No. of children	Total no.	% (95% CI)		No. of children	Total no.	% (95% CI)
Sensitivity	131	441	29.7 (25.5 to 34.2)		261	441	59.2 (54.4 to 63.8)		317	441	71.9 (67.4 to 76.0)
Specificity	1072	1075	99.7 (99.2 to 99.9)		1040	1075	96.7 (95.5 to 97.7)		979	1075	91.1 (89.2 to 92.7)
Positive predictive value	131	134	97.8 (93.6 to 99.5)		261	296	88.2 (83.9 to 91.6)		317	413	76.8 (72.4 to 80.7)
Negative predictive value	1072	1382	77.6 (75.3 to 79.7)		1040	1220	85.2 (83.1 to 87.2)		979	1103	88.8 (86.7 to 90.6)

BMI: body mass index; CI: confidence interval; SD: standard deviation.

**^a^** We calculated values as follows: sensitivity: [true positives/(true positives + false negatives)]; specificity: [true negatives/(true negatives + false positives)]; positive predictive value: [true positives/(true positives + false positives)]; negative predictive value: [true negatives/(true negatives + false negatives)].

^b^ We calculated the optimal cut-off z-score from the receiver operating characteristic curve (area under the curve: 0.86).

**Fig. 3:**
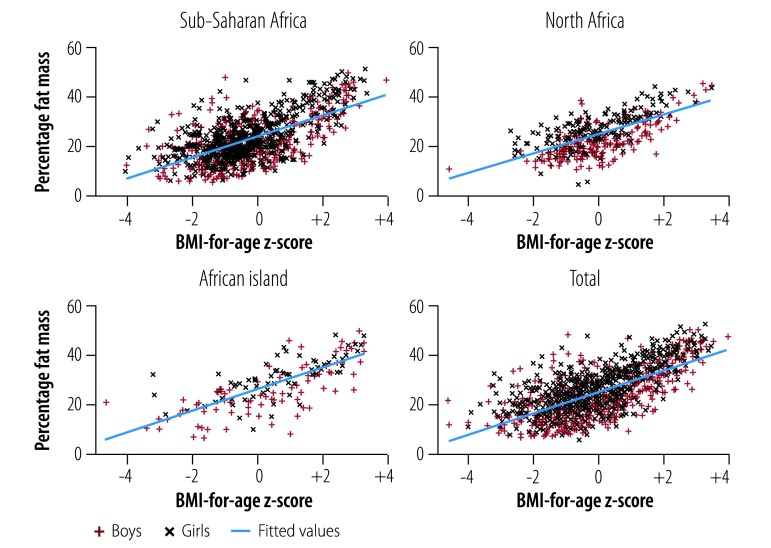
Receiver operator characteristic analysis of the ability of body mass index-for-age z-score to identify children with excessive fatness in eight African countries, 2013–2017

The receiver operator characteristic analysis is shown in Fig. 3. The optimal cut-off point in the BMI-for-age distribution for classifying excessive fatness was a BMI z-score of +0.58 SD (Table 4). At this cut-off the area under the curve was 0.86, sensitivity was 71.9% (95% CI: 67.4 to 76.0), specificity 91.1% (95% CI: 89.2 to 92.7), positive predictive value 76.8% (95% CI: 72.4 to 80.7) and negative predictive value 88.8% (95% CI: 86.7 to 90.6).

## Discussion

The present study has established the extent to which the WHO BMI-for-age definition of obesity underestimates the prevalence of excessive fatness in African children. Excessive fatness was present in nearly a third of children, suggesting that urban African environments are now highly obesogenic even for children. Excessive fatness was over three times more common than the prevalence of BMI-defined obesity. This difference is large enough to be meaningful for public health. For example, the case for policy action to prevent and control obesity is much weaker at an apparent prevalence of around 8% (based on BMI-for-age z-score > +2.00 SD in the present study) than at the prevalence of around 30% (excessive fatness) observed. To improve the estimation of prevalence, cut-off points in the BMI distribution lower than the z-score of +2.00 SD might be considered. At BMI z-score > +1.00 SD the ability to identify over-fatness was improved but not optimized. The optimal BMI z-score cut-off for classifying excessive fatness (which maximized the area under the curve) in our study was +0.58 SD.

There are no directly comparable studies in African children, or using the WHO-recommended definition based on BMI, but in non-African populations biases have been reported for other BMI-based definitions of obesity^9^^,^^10^ The present study adds to previous studies suggesting that underestimation of excessive fatness by BMI-for-age criteria is likely to be a global cause for concern.^19^^,^^20^ Our study shows that a high proportion of African children with apparently healthy BMI-for-age have excessive body fatness. The bias observed is unlikely to be due to a high body fat percentage secondary to unusually low fat-free mass (lean body mass). This is because of the consistency between the findings of the present study and studies for other populations.^9^^,^^10^ Furthermore, median fat mass index values, which measure fatness relatively independent of fat-free mass,^21^ were high in the present study. Reference data for fat mass index from British children of the same age (and measured in 2001, long after the childhood obesity epidemic had affected children in the United Kingdom of Great Britain and Northern Ireland) were very similar to those in the present study: 50th centile of 3.4 kg/m^2^ for boys and 4.2 kg/m^2^ for girls compared with 3.25 kg/m^2^ for boys and 3.91 kg/m^2^ for girls in the present study.^22^ Our findings are consistent with the evidence that body fatness of contemporary children is higher, across the range of body fatness, than that of children in the recent past.^23^^–^^25^

 The main strengths of the present study were the large sample size and narrow age range of the sample; the novelty of using the WHO BMI-for-age definition in an African setting; the novelty and value of having an unbiased definition of body fatness; the use of the Standards for Reporting of Diagnostic Accuracy Studies guidance^15^ in both the conduct and reporting of the study; and the standardization and quality control of both the study measurement methods and data management. A key limitation of the study was that we were unable to test definitively for differences in the diagnostic accuracy of BMI-for-age across different populations of African children. Our exploratory comparison of country groups by sub-Saharan Africa, North Africa and an island population was underpowered. A further limitation is generalizability. The participant age range of the present study limits our conclusions to 8 to 11 year olds, although our findings are consistent with those reported for younger and older participants, including adults, in systematic reviews of studies from non-African populations.^6^^,^^9^^,^^10^

In conclusion, excessive fatness is now prevalent among urban populations of African children and is likely to have serious future public health implications.^1^ While at a group level the BMI z-score and body fatness were related, BMI-for-age substantially underestimated the scale of the problem of excessive fatness and so may hinder or delay future obesity prevention and control efforts in Africa. Further research is needed to determine whether the sensitivity of the BMI-for-age indicator is especially low in African children compared with other populations. 
